# Evaluation of seven optical clearing methods in mouse brain

**DOI:** 10.1117/1.NPh.5.3.035007

**Published:** 2018-08-25

**Authors:** Peng Wan, Jingtan Zhu, Jianyi Xu, Yusha Li, Tingting Yu, Dan Zhu

**Affiliations:** a Huazhong University of Science and Technology, Britton Chance Center for Biomedical Photonics, Wuhan National Laboratory for Optoelectronics, Wuhan, Hubei, China; b Huazhong University of Science and Technology, MoE Key Laboratory for Biomedical Photonics, Collaborative Innovation Center for Biomedical Engineering, School of Engineering Sciences, Wuhan, Hubei, China

**Keywords:** tissue optical clearing, mouse brain, clearing capability, size change, fluorescence retention, imaging depth

## Abstract

Recently, a variety of tissue optical clearing techniques have been developed to reduce light scattering for imaging deeper and three-dimensional reconstruction of tissue structures. Combined with optical imaging techniques and diverse labeling methods, these clearing methods have significantly promoted the development of neuroscience. Each of them has its own characteristics with certain advantages and disadvantages. Though there are some comparison results, the clearing methods covered are limited and the evaluation indices lack uniformity, which made it difficult to select a best-fit protocol from numerous methods for clearing in practical applications. Hence, it is necessary to systematically assess and compare these clearing methods. We evaluated the performance of seven typical clearing methods, including 3-D imaging of solvent-cleared organs (3DISCO), ultimate DISCO (uDISCO), see deep brain (SeeDB), Sca*l*eS, $Clear^{T2}$, clear, unobstructed brain imaging cocktails and computational analysis, and passive CLARITY technique (PACT), on mouse brain samples. First, we compared the clearing effect and clearing time as well as size deformation on brain tissues. Further, we evaluated the fluorescence preservation and the increase of imaging depth induced by different methods. The results showed that 3DISCO, uDISCO, and PACT possessed excellent clearing capability on mouse brains, Sca*l*eS and SeeDB rendered moderate transparency, whereas $Clear^{T2}$ performed the worst. uDISCO and 3DISCO induced substantial size reduction on brain sections, and PACT expanded the mouse brain most seriously. Among those methods, Sca*l*eS performed best on fluorescence retention, 3DISCO induced the biggest decline of the fluorescence. PACT achieved the highest increase of imaging depth, and SeeDB and $Clear^{T2}$ possessed the shallowest imaging depth. This study is expected to provide important reference for users in choosing the most suitable brain optical clearing method.

## 1 Introduction

Three-dimensional (3-D) imaging of intact brain is indispensable for high-resolution mapping of neuronal networks, which is valuable for understanding brain structural–functional relationships.^1^^–^^3^ Except for the widespread histological sectioning methods and emerging automated serial-sectioning and imaging approaches, optical imaging techniques make the 3-D imaging of thick brain tissues possible via optical sectioning with no need of thin slicing,^4^^–^^6^ such as confocal microscopy, two-photon microscopy, light-sheet microscopy, and so on. However, the imaging depth of these microscopies suffers from strong light scattering of brain tissues.^1^^,^^7^^,^^8^

Tissue optical clearing method has been proposed to address this issue. In the past decade, various clearing methods have been developed to tansparentize large-volume brain tissues, using physical or chemical strategies.^9^^–^^35^ These clearing methods can be roughly divided into two categories, including the solvent-based and the aqueous-based clearing methods. The former category includes benzyl alcohol and benzyl benzoate (BABB),^11^ 3-D imaging of solvent-cleared organs (3DISCO),^12^^–^^14^ immunolabeling-enabled three-dimensional imaging of solvent-cleared organs (iDISCO), ^15^ ultimate DISCO (uDISCO),^16^ and so on, usually goes through dehydration, lipid removal and refractive index matching with reagents.^11^^–^^16^ The latter category can further be divided into three types: simple immersion, such as see deep brain (SeeDB),^18^ SeeDB2,^19^ FRUIT (a method based on fructose and urea),^20^ ${\mathrm{Clear}}^{T2}$ (a detergent- and solvent-free clearing method);^21^ hyperhydration, such as Sca*l*e (an aqueous reagent that renders biological samples transparent),^22^ Sca*l*eS (a sorbitol-based Sca*l*e),^23^ clear, unobstructed brain imaging cocktails and computational analysis (CUBIC),^24^^–^^26^ CUBIC based on transcardial perfusion (CB-perfusion);^24^^,^^25^ and hydrogel embedding, such as clear lipid-exchanged acrylamide-hybridized rigid imaging/immunostaining/*in situ* hybridization-compatible tissue-hYdrogel (CLARITY),^27^^,^^28^ passive CLARITY technique (PACT),^29^^–^^31^ perfusion-assisted agent release *in situ* (PARS),^29^^,^^30^ system-wide control of interaction time and kinetics of chemicals (SWITCH),^32^ CLARITY-TDE (2, 2’-thiodiethanol),^33^ and so on.

These clearing methods have provided essential tools for mapping brain wiring diagrams and greatly promoted the development of neuroscience.^2^^,^^3^ They were usually developed for certain application scopes and had their own advantages and disadvantages. In practical experiments, it is necessary but difficult to select the best-fit method from numerous methods. Though there are some comparison results in recently published papers,^16^^,^^18^^,^^23^ the clearing methods covered are limited and the evaluation indices are lack of uniformity, which makes it rather difficult to make an appropriate selection. Hence, a comprehensive and systematic assessment of various types of optical clearing methods is in great request.

In this work, we chose seven clearing methods, including uDISCO, 3DISCO, SeeDB, Sca*l*eS, CUBIC, $Clear^{T2}$, and PACT,^12^^–^^14^^,^^16^^,^^18^^,^^21^^,^^23^^–^^26^^,^^29^^–^^31^ and compared their clearing performance from different aspects. First, we evaluated their clearing capability based on the transparency and clearing time for both brain sections and whole brains. We also assessed the size change by calculating the shrinkage or expansion ratio of brain tissues. Then, we quantitatively compared the retention of green fluorescent protein (GFP) and the increase of imaging depth for adult mouse brain tissues. This work can provide a reference for the selection of optical clearing methods in practical applications.

## 2 Methods

### 2.1 Preparation of Samples

The animals used in this study include *Thy1*-GFP-M mice and *CX3CR1*-GFP mice (9- to 13-week-old). Mice were deeply anesthetized with a mixture of 2% α-chloralose and 10% urethane (8  ml/kg) through intraperitoneal injection. Then, they were transcardially perfused with 0.01M phosphate-buffered saline (PBS) (Sigma) followed by 4% paraformaldehyde (PFA) (Sigma-Aldrich). The mouse brains were dissected and postfixed overnight in 4% PFA at 4°C. The brains were sliced into 2-mm-thick coronal sections with a vibratome (Leica VT 1000 s). The animal care and experimental protocols were in accordance with the Experimental Animal Management Ordinance of Hubei Province, China and the guidelines from the Huazhong University of Science and Technology and have been approved by the Institutional Animal Ethics Committee of Huazhong University of Science and Technology.

### 2.2 Clearing Protocols

In this work, we selected different kinds of tissue optical clearing methods, including the solvent-based clearing methods, such as 3DISCO and uDISCO, and the aqueous-based clearing methods, such as SeeDB and $Clear^{T2}$ based on simple immersion, CUBIC and Sca*l*eS based on hyperhydration, and PACT based on hydrogel embedding. All the clearing protocols in this work were performed by referring to the previous literatures.^12^^–^^14^^,^^16^^,^^18^^,^^21^^,^^23^^–^^26^^,^^29^^–^^31^ Sca*l*eS and PACT were conducted with slight adjustments as described in the following.

For standard Sca*l*eS protocol, after incubated in Sca*l*eS0 solution, the samples were successively immersed in Sca*l*eS1, Sca*l*eS2, and Sca*l*eS3 solutions. After washing with PBS, samples were finally incubated in Sca*l*eS4. All steps were conducted at 37°C except PBS washing (4°C). Each step took 12 h for 2-mm-thick brain sections and 24 h for whole brains. However, the N-acetyl-*L*-hydroxyproline, as an ingredient in Sca*l*eS0, must be from Skin Essential Active (Taiwan), was hard to obtain. Considering the N-acetyl-*L*-hydroxyproline is not the primary ingredient for clearing, we excluded it from Sca*l*eS0 in this work. For rapid Sca*l*eSQ(5) protocol, 2-mm-thick brain sections were incubated in Sca*l*eSQ(5) for 2 h at 37°C and then Sca*l*eS4(0) for 2 h at room temperature with slight shaking. For PACT, the sample-hydrogel solution was degassed with injection syringe by neglecting nitrogen infusion, followed by incubating in 8% SDS solution (prepared with 0.01M PBS) with slight shaking.

### 2.3 Measurement of Light Transmittance

Commercially available spectrophotometer (Lambda 950, PerkinElmer) was used to measure the transmittance of brain sections and whole brains.

### 2.4 Imaging

Digital camera (HDC-HS900GK) was used to acquire the bright-field images of samples. Confocal fluorescence microscopy (LSM710, Zeiss, Germany), equipped with the Fluar 10× /0.5 objective (dry, working distance 2.0 mm) and Plan-Apochromat 20× /0.8 objective (dry, working distance 0.55 mm), was used to acquire the GFP fluorescence images of brain sections. Before and after clearing, the fluorescence images were obtained under the same imaging parameters.

### 2.5 Data Analysis

We used ImageJ software and MATLAB for image processing and quantitative analysis of data. The analysis was derived from the literature.^34^ For size change, the samples were outlined and the areas were measured with ImageJ software, and the linear size change was quantified by dividing the area of cleared samples by the area of uncleared samples in PBS and taking the square root of the ratio.

For fluorescence quantification, the mean intensity of fluorescence of same neurons in the cortex of coronal brain sections was measured before clearing (supposed to be “A”) and after clearing (supposed to be “B”), and the relative mean intensity of fluorescence was calculated as “B/A.” The total intensity of fluorescence was calculated by multiplying the mean intensity of fluorescence by sample area, and the relative total intensity of fluorescence was calculated as similar as mean intensity.

The imaging depth was calculated based on the contrast decay by referring to the literatures.^33^^,^^35^ It should be noted that the contrast-to-noise ratio (CNR),^36^ as a good representative to measure the depth, was not used because of the inaccurate segmentation due to the small size and specific structure of the objects of interest (i.e., microglia). Here, the imaging contrast was obtained according to Eq. (1). Where I represents the grayscale value for each pixel, $I_{\text{mean}}$ indicates the average intensity of the image, and n is the number of total pixels. The imaging depth is determined where the contrast drops to 1/2 of the maximum from where it rises 1/2 of the maximum

$$\text{contrast}=\sqrt{\frac{\sum {\left(I-I_{\text{mean}}\right)}^{2}}{n-1}}.$$ (1)

## 3 Results

### 3.1 Comparison of Clearing Capability

**Fig. 1:**
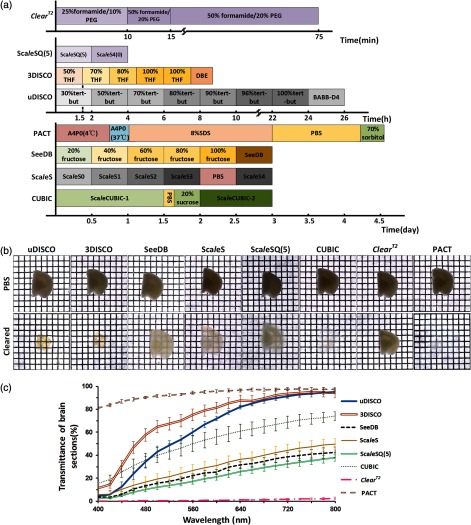
Comparison of clearing capability of seven clearing methods for 2-mm-thick brain sections. (a) Clearing protocols of different clearing methods for 2-mm-thick brain sections. PEG, polyethylene glycol 8000; THF, tetrahydrofuran; DBE, dibenzyl ether; tert-but, tert-butanol; A4P0, 4% acrylamide in PBS with 0.25% VA-044; SDS, sodium dodecyl sulfate. (b) Bright-field images of 2-mm-thick adult mouse brain sections before and after clearing. Grid size, 1.45  mm×1.45  mm. (c) Transmittance curves of brain sections cleared with various clearing methods. n=3 brain sections for each group.

To compare the clearing capability of various optical clearing methods, we selected seven clearing methods (uDISCO, 3DISCO, SeeDB, Sca*l*eS, CUBIC, $Clear^{T2}$, and PACT), and cleared 2-mm-thick *Thy1*-GFP-M brain sections with these methods, respectively. Figure 1(a) shows the time of each treatment step of seven clearing protocols. The samples incubated in the clearing solutions were put on the grid paper, and the transmittance images were photographed. The optical transparency of the 2-mm-thick mouse brain slices before and after clearing is shown in Fig. 1(b). Figure 1(c) displays the transmittance of brain sections for each clearing method. uDISCO, 3DISCO, CUBIC, and PACT show excellent clearing capability on brain sections, and the organic-solvent based methods (uDISCO and 3DISCO) cost short time with 9 hours to 1 day, whereas CUBIC and PACT take longer time with 3 to 4.5 days. SeeDB and Sca*l*eS show average level of clearing capability on brain sections, and they also take a long time, about 3 days. Sca*l*eSQ(5), as a submethod of Sca*l*eS, is a rapid clearing protocol specific for brain sections. It shows a certain degree of transparency with rather short time (4 h). Though the $Clear^{T2}$ method costs the shortest time, its clearing capability is the worst.

**Fig. 2:**
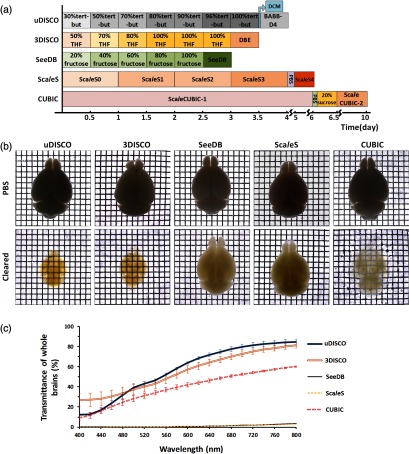
Comparison of clearing capability of five whole-brain clearing methods. (a) Clearing protocols of different clearing methods for whole brains. DCM, dichloromethane. (b) Bright-field images of adult mouse brains before and after clearing with five whole-brain clearing methods. Grid size, 1.45  mm×1.45  mm. (c) Transmittance curves of whole brains cleared with various clearing methods. n=3 brains for each group.

As claimed in the original papers of these methods, uDISCO, 3DISCO, SeeDB, Sca*l*eS, and CUBIC could render whole mouse brain transparent with simple incubation. Here, we cleared the adult whole brains with these five methods and evaluated their clearing capability for large-volume tissues. Figure 2(a) displays the schedules of the clearing methods for whole brains. Figure 2(b) shows the optical transparency of the brains before and after clearing. Figure 2(c) displays the transmittance of whole brains for each clearing method. In terms of the whole-brain clearing, uDISCO and 3DISCO show best clearing capability by taking about 4 days. CUBIC takes the second place but requires longest processing time (∼10 days). In addition, uDISCO and 3DISCO can achieve the homogeneous clearing of all brain regions, whereas CUBIC does not demonstrate good clearing effect in the myelinated areas as the other regions. Sca*l*eS demonstrates a weak clearing capability even taking a long time (6 days). While the optical transparency of SeeDB is the weakest though the time it takes is the shortest.

### 3.2 Size Changes

**Fig. 3:**
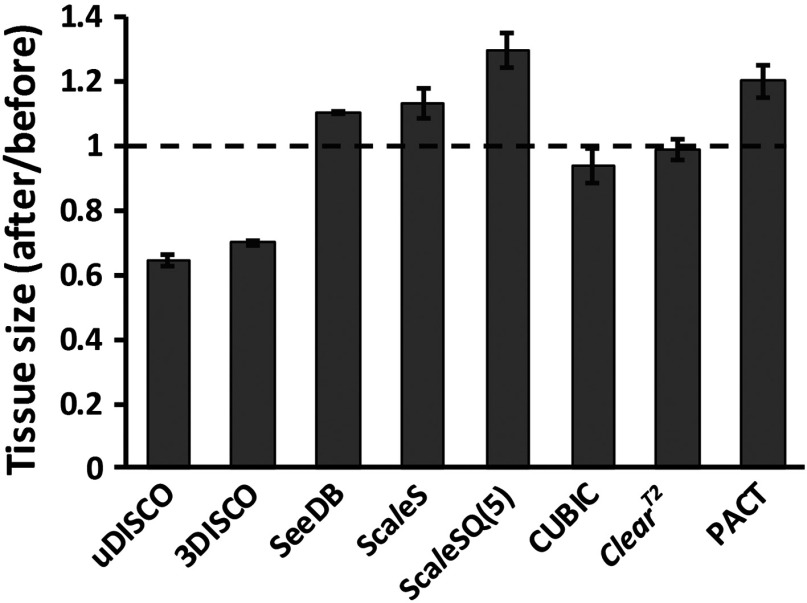
Quantitative comparison of sample deformation. Quantification of the linear change for 2-mm-thick adult *Thy1*-GFP-M mouse brain sections after various clearing methods clearing (Error bars denote standard deviations; n=3 brain sections for each group).

The size change is another important criterion for evaluating the clearing methods. We calculated the linear changes of 2-mm-thick sections cleared by seven optical clearing methods. Figure 3 shows that uDISCO and 3DISCO induce substantial size reduction on mouse brain. They reduce the size of brain sections up to 30% to 35%. CUBIC and $Clear^{T2}$ slightly shrink the samples (<7%). Whereas, SeeDB and Sca*l*eS have slight expansion effect on samples, they expand the brain sections about 10% to 12%. PACT and Sca*l*eSQ(5) expand the mouse brains by 20% to 30%.

### 3.3 Retention of GFP Fluorescence

**Fig. 4:**
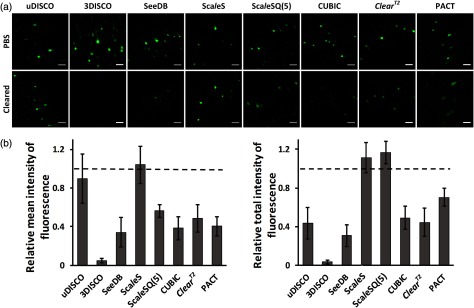
Retention of GFP fluorescence. (a) Confocal imaging of cortical neurons in *Thy1*-GFP-M mouse brains before and after various methods clearing. Each image is a maximum projection of image stacks (40 to 60  μm). Scale bar, 50  μm. (b) The relative changes of mean intensity of fluorescence and total intensity of fluorescence after uDISCO, 3DISCO, SeeDB, Sca*l*eS, and Sca*l*eSQ(5), CUBIC, $Clear^{T2}$, and PACT clearing (Error bars denote standard deviations; n=15, 14, 15, 13, 17, 17, 13, and 21 neurons, respectively).

The retention of GFP fluorescence is critical for 3-D imaging of tissue structures. We imaged the neurons of adult *Thy1*-GFP-M mouse brain cortex before and after clearing with different methods, and then calculated the intensity of GFP fluorescence. Figure 4(a) shows the maximum projection of z-stack images (40 to 60  μm) before and after clearing, and Fig. 4(b) shows the relative fluorescence intensity of samples. For relative mean intensity of fluorescence, Sca*l*eS and uDISCO show the best GFP fluorescence intensity, and followed by Sca*l*eSQ(5), $Clear^{T2}$, PACT, CUBIC, and SeeDB. While 3DISCO shows the lowest fluorescence intensity. The relative mean intensity of fluorescence is determined by not only the chemical influence of clearing agents but also the physical influence such as tissue shrinkage or expansion. Hence, we also compared the relative total intensity of fluorescence of different methods by taking size change into consideration. The results demonstrate that Sca*l*eSQ(5) and Sca*l*eS preserve the GFP information best, and followed by PACT retaining 70% of the fluorescence. CUBIC, uDISCO, and $Clear^{T2}$ quench about half the fluorescence (44% to 50%). SeeDB quenches the fluorescence more seriously. 3DISCO induces the biggest decline of the fluorescence.

### 3.4 Improvement of Imaging Depth

**Fig. 5:**
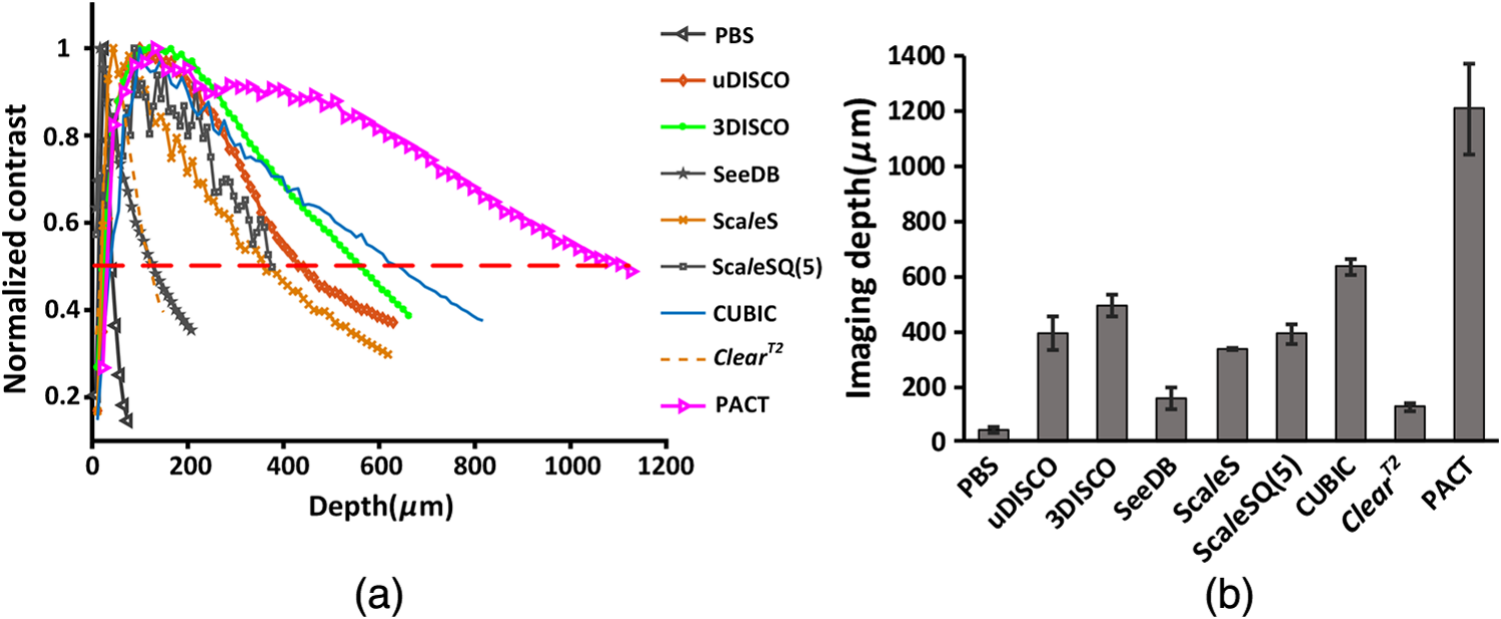
Quantification of imaging depth with different clearing methods. (a) Image contrast for 2-mm-thick *CX3CR1*-GFP mouse brain sections before (in PBS) and after various methods clearing. The imaging depth is determined where the contrast drops to 1/2 of the maximum from where it rises 1/2 of the maximum. (b) Quantitative data of imaging depth before (in PBS) and after clearing with various clearing protocols (Error bars denote standard deviations; n=6, 3, 3, 3, 3, 3, 3, 3, and 3 brain sections, respectively).

The purpose of optical clearing methods is to improve the imaging depth for imaging deeper structural information. In this study, the imaging depth is calculated based on contrast decay. Figure 5(a) shows the image contrast for 2-mm-thick *CX3CR1*-GFP brain sections before and after clearing with different methods. Figure 5(b) is the quantitation of imaging depth for different optical clearing methods. For uncleared samples in PBS, the imaging depth are about 40 to 50  μm. After clearing, their imaging depth obviously increase. PACT achieves the deepest imaging depth (∼1200  μm), and followed by CUBIC, 3DISCO, and uDISCO, their imaging depth are >400  μm. The imaging depth of Sca*l*eSQ(5) and Sca*l*eS are about 350  μm. SeeDB and $Clear^{T2}$ possess the shallowest imaging depth (<200  μm).

## 4 Discussion

In this work, we make a systemic comparison of seven clearing methods, including uDISCO, 3DISCO, SeeDB, Sca*l*eS, CUBIC, $Clear^{T2}$, and PACT. We evaluated their clearing capability by comparing the transparency and clearing time for both brain sections and whole brains. Then, we did the quantitative comparison of sample deformation by calculating the shrinkage or expansion ratio of brain tissues. We also evaluated the fluorescence retention and the increase of imaging depth. The results are summed up in Table 1 and can provide references for users in choosing suitable brain optical clearing method.

**Table 1:** Comparison of various tissue clearing methods.

Method	Clearing capability	Time to clear	Size change	Fluorescence signal^a^	Imaging depth	Chemical permeability^b^
uDISCO	Excellent	One day-days	Strong shrinkage	Preserved	Very deep	—
3DISCO	Excellent	Hours-days	Strong shrinkage	Major loss	Very deep	—
SeeDB	Medium	Days	Slight expansion	Modest loss	Shallow	—
Sca*l*eS	Medium	Days	Slight expansion	Preserved	Deep	2 mm/4 days^c^
Sca*l*eSQ(5)	Weak	Hours	Strong expansion	Modest loss	Deep	—
CUBIC	Good	Days-weeks	Slight shrinkage	Modest loss	Very deep	3.2 mm/6 days^d^
${\text{Clear}}^{T2}$	Weak	Hours	Slight shrinkage	Modest loss	Shallow	—
PACT	Excellent	Days	Strong expansion	Modest loss	Very deep	3 mm/7 days

Note: The samples are from 2-mm-thick brain sections to whole brain. The minimum time to clear represents the time for clearing of 2-mm-thick brain sections; and the maximum time to clear is the time for clearing of whole brains. “—,” indicates that the clearing protocol would not enhance the antibody penetration and the immunostaining was generally conducted prior to the clearing protocol.

a Mean intensity of GFP fluorescence.

b Not tested (N.T.).

c Data of AbSca*l*e protocol.

d Data for staining hypothalamus.

As mentioned above, the clearing methods evaluated in this study include both the solvent-based and aqueous-based protocols. From the results, we can see that the solvent-based clearing protocols, such as 3DISCO and uDISCO, can achieve highest level of brain transparency and substantial sample shrinkage within reasonable time. These are remarkable advantages for imaging large-volume specimens combined with light-sheet microscopy. However, 3DISCO has the major limitation of fast quenching of endogenous fluorescence signal. While uDISCO demonstrates obviously better fluorescence preserving capability and can overcome this problem to be readily used in many biological researches, as described in the original literature.^16^

It should be noted that SeeDB, a method based on simple immersion in graded fructose solutions, has limited clearing capability and is only suitable for small samples, just like $Clear^{T2}$. It has been claimed that SeeDB can preserve the YFP fluorescence well, and here we found that the GFP fluorescence decreased after SeeDB, as some other studies showed.^16^^,^^18^^,^^37^ With the hyperhydration effect of urea, CUBIC and Sca*l*eS achieve obviously higher tissue transparency than SeeDB on brain slices. However, the high concentration of Triton X-100 used in CUBIC resulted in a decrease of GFP fluorescence. Sca*l*eS restricts the use of lipid detergent, so it preserves the GFP fluorescence well.^1^^,^^23^^,^^24^ PACT demonstrates an excellent clearing capability for samples; however, due to the use of strong lipid detergent (8% SDS), it induces modest loss of GFP fluorescence.^29^^,^^30^ In practical experiments, the clearing effect and fluorescence preserving capability should be both taken into accounts for choosing suitable methods. In addition, the condition of clearing procedures should be adjusted according to specific experimental needs.

It has been stated that the use of a noncorrected air objective introduces severe spherical aberrations, which not only degrades resolution but also reduces the peak value of the point spread function.^38^ In adaptive optics, the amount of this reduction is termed “Strehl ratio,” and its effect is that objects are usually dimmer when observed through an aberrated instrument. Since the aberration depends on the refractive index of the clearing solution, imaging in different clearing agents leads to different Strehl ratios and thus to different image intensities. For instance, organic solvents have very high refractive indices and therefore introduce stronger aberrations when using an air objective. The poorer “fluorescence performance” of organic solvents might thus have also optical causes, in addition to chemical ones.

In consideration of the differences in size changes, we measured both the relative mean intensity of fluorescence and relative total intensity of fluorescence of neurons for comparison. For shrinkage, the fluorescent proteins in neurons come closer and become denser, whereas for expansion, it is just the reverse. Hence, the relative mean intensity of fluorescence depends on not only the chemical influence of clearing agents but also the physical influence such as tissue shrinkage or expansion. We cannot simply determine the fluorescence decrease as the quenching of fluorescent proteins by neglecting the size changes. It is recommended to measure both parameters to give a comprehensive evaluation of fluorescence signal.

As light is attenuated inside tissue, the deeper it goes, the lower the signal. Both absorption and scattering would affect the penetration, but only scattering blurs the image. The CNR^36^ is a good representative to measure the depth. However, for the images in this study, the CNR keeps the value above 2 and even increases at the deep depth with decrease of both mean signal intensity and noise for some groups, hence the CNR calculation cannot be used for quantification of imaging depth. This might be due to the small size and specific structure of the objects of interest (i.e., microglia), leading to the inaccurate segmentation. So, we used the decay of the image contrast value for evaluation of the imaging depth, which has been used by some researchers in published papers.^33^^,^^35^ Though this is not a good representative to measure imaging depth, it is a compromise choice for comparison study of different clearing methods.

In general, the imaging depth is relative to both tissue transparency and fluorescence intensity. Here, we imaged the neurons in brain cortex regions of coronal sections and calculated the imaging depth based on contrast decay of image stacks. The transparency induced by most clearing methods are roughly homogeneous except Sca*l*eSQ(5). The inhomogeneity of transparency in Sca*l*eSQ(5) is supposed to be due to its quick clearing protocol and the differences of tissue components in different brain regions. For example, the myelin-rich middle area is more difficult to clear than the cortex area.

Some features that we have quantified are inherently intertwined in the tissue, such as fluorescence intensity and contrast. Less scattering will result in both higher contrast and higher fluorescence since fluorophores are excited more efficiently and fluorescence light is more efficiently collected. However, the detected fluorescence intensity is not only related to the optical scattering but also the chemical quenching of fluorescence induced by each protocol. Hence, we quantified the fluorescence intensity at the superficial area of brain sections and normalized the contrast of images for comparison.

For some of the clearing methods, such as 3DISCO, uDISCO, and SeeDB, the immunostaining or labeling is usually conducted prior to clearing, which has no effect of increasing chemical permeability due to lack of tissue loosening. While some other clearing methods, such as Sca*l*eS, CUBIC, and PACT, not only reduce the optical scattering but also increase the chemical permeability that provides better labeling for fluorescence imaging. The brain can be immunostained across these three clearing methods with similar penetration rate of conventional antibodies (Table 1).

It is worth noting that the optical clearing methods mentioned in this paper are limited to fixed samples, thus are not applicable to *in vivo* imaging and live brain/brain slices imaging. A recent work reported a nontoxic medium, iodixanol, as the tunable refractive index matching in live specimens,^39^ but it is challenging to realize imaging in whole brain. While photoacoustic computed tomography has demonstrated the whole-brain imaging without clearing and even *in vivo* by detecting the light-induced ultrasound,^40^^–^^43^ providing powerful tools for both anatomical and functional whole brain imaging.

CLARITY is a good representative of optical clearing method based on hydrogel embedding, and there are many variants of this method, such as PACT,^29^ PARS,^29^ Bone-CLARITY (a specialized CLARITY protocol for bone tissues),^44^ a plant-enzyme-assisted CLARITY protocol for plant tissues (PEA-CALRITY),^45^ and so on, that have been widely used. It had been mentioned in the literature^29^ that CLARITY in its original form used electrophoretic tissue clearing to extract lipids from large samples, which can be challenging to implement and can cause variability in final tissue quality. Hence, we used PACT, a representative hydrogel-embedding method for passive lipid extraction of 1- to 3-mm-thick tissues, for comparison due to its applicability and ease of handling in this work. In addition, the evaluation on some other tissue samples, such as liver, kidney, and so on, needs further research. Except the five parameters used in this work, more evaluation standards such as compatibility with different fluorescent probes deserve to be investigated in the future.

## 5 Conclusion

In this study, we made a systematical evaluation for various optical clearing methods, including uDISCO, 3DISCO, SeeDB, Sca*l*eS, CUBIC, $Clear^{T2}$, and PACT. The evaluation contains clearing capability, size deformation, clearing speed, fluorescence retention, and imaging depth. For clearing capability and speed, the uDISCO, 3DISCO, CUBIC, and PACT methods possess excellent clearing capability, uDISCO and 3DISCO cost short time, whereas CUBIC and PACT take long time. For size change, the uDISCO and 3DISCO methods have strong shrinkage effect on brain sections, and PACT expands the mouse brain seriously, whereas other methods change the brain size slightly. For fluorescence retention, Sca*l*eS preserves the GFP fluorescence signal best, followed by $Clear^{T2}$, PACT, CUBIC, and SeeDB; and 3DISCO quenches almost all the fluorescence. For imaging depth, PACT has the deepest imaging depth, followed by CUBIC, 3DISCO, and uDISCO. The imaging depth of Sca*l*eS is intermediate level. While SeeDB and $Clear^{T2}$ possess the shallowest imaging depth. This study can provide an important reference for users in selecting suitable optical clearing method in brain samples.

## References

[1] Richardson D. S. Lichtman J. W., “Clarifying tissue clearing,” Cell 162(2), 246–257 (2015). CELLB5 0092-8674 10.1016/j.cell.2015.06.067 26186186 PMC4537058

[r2] Oh S. W. et al., “A mesoscale connectome of the mouse brain,” Nature 508, 207–214 (2014). 10.1038/nature13186 24695228 PMC5102064

[3] Arthur W. T. et al., “Postmortem cryosectioning as an anatomic reference for human brain mapping,” Comput. Med. Imaging Graphics 21(2), 131–141 (1997). 10.1016/S0895-6111(96)00072-9 9152579

[4] Conchello J. A. Lichtman J. W., “Optical sectioning microscopy,” Nat. Methods 2(12), 920–931 (2005).1548-7091 10.1038/nmeth815 16299477

[r5] Mertz J., “Optical sectioning microscopy with planar or structured illumination,” Nat. Methods 8(10), 811–819 (2011).1548-7091 10.1038/nmeth.1709 21959136

[6] Reynaud E. G. et al., “Light sheet-based fluorescence microscopy: more dimensions, more photons, and less photodamage,” HFSP J. 2(5), 266–275 (2008). HJFOA5 1955-2068 10.2976/1.2974980 19404438 PMC2639947

[7] Tuchin V. V., “Tissue optics and photonics: light-tissue interaction,” J. Biomed. Photonics Eng. 1(2), 98–134 (2015). 10.18287/JBPE-2015-1-2-98

[8] Lichtman J. W. Conchello J. A., “Fluorescence microscopy,” Nat. Methods 2(12), 910–919 (2005).1548-7091 10.1038/nmeth817 16299476

[9] Lai H. M. et al., “Next generation histology methods for three-dimensional imaging of fresh and archival human brain tissues,” Nat. Commun. 9(1), 1066 (2018). NCAOBW 2041-1723 10.1038/s41467-018-03359-w 29540691 PMC5852003

[r10] Yu T. et al., “Quantitative analysis of dehydration in porcine skin for assessing mechanism of optical clearing,” J. Biomed. Opt. 16(9), 095002 (2011). JBOPFO 1083-3668 10.1117/1.3621515 21950911

[r11] Dodt H. U. et al., “Ultramicroscopy: three-dimensional visualization of neuronal networks in the whole mouse brain,” Nat. Methods 4(4), 331–336 (2007).1548-7091 10.1038/nmeth1036 17384643

[r12] Erturk A. et al., “Three-dimensional imaging of solvent-cleared organs using 3DISCO,” Nat. Protoc. 7(11), 1983–1995 (2012).1754-2189 10.1038/nprot.2012.119 23060243

[r13] Erturk A. et al., “Three-dimensional imaging of the unsectioned adult spinal cord to assess axon regeneration and glial responses after injury,” Nat. Med. 18(1), 166–171 (2012).1078-8956 10.1038/nm.2600 22198277

[r14] Becker K. et al., “Chemical clearing and dehydration of GFP expressing mouse brains,” PLoS One 7(3), e33916 (2012). POLNCL 1932-6203 10.1371/journal.pone.0033916 22479475 PMC3316521

[r15] Renier N. et al., “iDISCO: a simple, rapid method to immunolabel large tissue samples for volume imaging,” Cell 159(4), 896–910 (2014). CELLB5 0092-8674 10.1016/j.cell.2014.10.010 25417164

[r16] Pan C. et al., “Shrinkage-mediated imaging of entire organs and organisms using uDISCO,” Nat. Methods 13(10), 859–867 (2016).1548-7091 10.1038/nmeth.3964 27548807

[r17] Yu T. et al., “Optical clearing for multiscale biological tissues,” J. Biophotonics 11(2), e201700187 (2017). 10.1002/jbio.201700187 29024450

[r18] Ke M. T. Fujimoto S. Imai T., “SeeDB: a simple and morphology-preserving optical clearing agent for neuronal circuit reconstruction,” Nat. Neurosci. 16(8), 1154–1161 (2013). NANEFN 1097-6256 10.1038/nn.3447 23792946

[r19] Ke M. T. et al., “Super-resolution mapping of neuronal circuitry with an index-optimized clearing agent,” Cell Rep. 14(11), 2718–2732 (2016). 10.1016/j.celrep.2016.02.057 26972009

[r20] Hou B. et al., “Scalable and DiI-compatible optical clearance of the mammalian brain,” Front. Neuroanat. 9, 19 (2015). 10.3389/fnana.2015.00019 25759641 PMC4338786

[r21] Kuwajima T. et al., “ClearT: a detergent- and solvent-free clearing method for neuronal and non-neuronal tissue,” Development 140(6), 1364–1368 (2013). 10.1242/dev.091844 23444362 PMC3912244

[r22] Hama H. et al., “Scale: a chemical approach for fluorescence imaging and reconstruction of transparent mouse brain,” Nat. Neurosci. 14(11), 1481–1488 (2011). NANEFN 1097-6256 10.1038/nn.2928 21878933

[r23] Hama H. et al., “ScaleS: an optical clearing palette for biological imaging,” Nat. Neurosci. 18(10), 1518–1529 (2015). NANEFN 1097-6256 10.1038/nn.4107 26368944

[r24] Susaki E. A. et al., “Whole-brain imaging with single-cell resolution using chemical cocktails and computational analysis,” Cell 157(3), 726–739 (2014). CELLB5 0092-8674 10.1016/j.cell.2014.03.042 24746791

[r25] Tainaka K. et al., “Whole-body imaging with single-cell resolution by tissue decolorization,” Cell 159(4), 911–924 (2014). CELLB5 0092-8674 10.1016/j.cell.2014.10.034 25417165

[r26] Susaki E. A. et al., “Advanced CUBIC protocols for whole-brain and whole-body clearing and imaging,” Nat. Protoc. 10(11), 1709–1727 (2015).1754-2189 10.1038/nprot.2015.085 26448360

[r27] Tomer R. et al., “Advanced CLARITY for rapid and high-resolution imaging of intact tissues,” Nat. Protoc. 9(7), 1682–1697 (2014).1754-2189 10.1038/nprot.2014.123 24945384 PMC4096681

[r28] Chung K. et al., “Structural and molecular interrogation of intact biological systems,” Nature 497(7449), 332–337 (2013). 10.1038/nature12107 23575631 PMC4092167

[r29] Yang B. et al., “Single-cell phenotyping within transparent intact tissue through whole-body clearing,” Cell 158(4), 945–958 (2014). CELLB5 0092-8674 10.1016/j.cell.2014.07.017 25088144 PMC4153367

[r30] Treweek J. B. et al., “Whole-body tissue stabilization and selective extractions via tissue-hydrogel hybrids for high-resolution intact circuit mapping and phenotyping,” Nat. Protoc. 10(11), 1860–1896 (2015).1754-2189 10.1038/nprot.2015.122 26492141 PMC4917295

[r31] Yu T. et al., “Elevated-temperature-induced acceleration of PACT clearing process of mouse brain tissue,” Sci. Rep. 7, 38848 (2017). SRCEC3 2045-2322 10.1038/srep38848 28139694 PMC5282525

[r32] Murray E. et al., “Simple, scalable proteomic imaging for high-dimensional profiling of intact systems,” Cell 163(6), 1500–1514 (2015). CELLB5 0092-8674 10.1016/j.cell.2015.11.025 26638076 PMC5275966

[r33] Costantini I. et al., “A versatile clearing agent for multi-modal brain imaging,” Sci. Rep. 5, 9808 (2015). SRCEC3 2045-2322 10.1038/srep09808 25950610 PMC4423470

[r34] Yu T. et al., “Rapid and prodium iodide-compatible optical clearing method for brain tissue based on sugarsugar-alcohol,” J. Biomed. Opt. 21, 081203 (2016). JBOPFO 1083-3668 10.1117/1.JBO.21.8.081203 26968577

[35] Vlieg R. C. et al., “Evaluating different passive optical clearing protocols for two-photon deep tissue imaging in adult intact visceral and neuronal organs,” bioRxiv (2015). 10.1101/018622

[36] Song X. M. et al., “Automated region detection based on the contrast-to-noise ratio in near-infrared tomography,” Appl. Opt. 43, 1053–1062 (2004). APOPAI 0003-6935 10.1364/AO.43.001053 15008484

[37] Fretaud M. et al., “High-resolution 3D imaging of whole organ after clearing: taking a new look at the zebrafish testis,” Sci. Rep. 7, 43012 (2017). SRCEC3 2045-2322 10.1038/srep43012 28211501 PMC5314416

[38] Silvestri L. Sacconi L. Pavone F. S., “Correcting spherical aberrations in confocal light sheet microscopy: a theoretical study,” Microsc. Res. Tech. 77(7), 483–491 (2014). MRTEEO 1059-910X 10.1002/jemt.v77.7 24395714

[39] Boothe T. et al., “A tunable refractive index matching medium for live imaging cells, tissues and model organisms,” Elife 6, e27240 (2017). 10.7554/eLife.27240 28708059 PMC5582871

[40] Wang X. et al., “Three-dimensional laser-induced photoacoustic tomography of mouse brain with the skin and skull intact,” Opt. Lett. 28(19), 1739–1741 (2003). OPLEDP 0146-9592 10.1364/OL.28.001739 14514085

[r41] Li L. et al., “Label-free photoacoustic tomography of whole mouse brain structures ex vivo,” Neurophotonics 3(3), 035001 (2016). 10.1117/1.NPh.3.3.035001 29181425 PMC5696384

[r42] Li L. et al., “Single-impulse panoramic photoacoustic computed tomography of small-animal whole-body dynamics at high spatiotemporal resolution,” Nat. Biomed. Eng. 1(5) 0071 (2017). 10.1038/s41551-017-0071 29333331 PMC5766044

[43] Li L. et al., “Linear-array based full-view high-resolution photoacoustic computed tomography of whole mouse brain functions in vivo,” Proc. SPIE 10494, 104941H (2018). PSISDG 0277-786X 10.1117/12.2289118

[44] Greenbaum A. et al., “Bone CLARITY: clearing, imaging, and computational analysis of osteoprogenitors within intact bone marrow,” Sci. Transl. Med. 9, eaah6518 (2017). STMCBQ 1946-6234 10.1126/scitranslmed.aah6518 28446689

[45] Palmer W. M. et al., “PEA-CLARITY: 3D molecular imaging of whole plant organs,” Sci. Rep. 5, 13492 (2015). SRCEC3 2045-2322 10.1038/srep13492 26328508 PMC4556961

[46] Wan P. et al., “Comparison of seven optical clearing methods for mouse brain,” Proc. SPIE 10481, 104811I (2018). PSISDG 0277-786X 10.1117/12.2289392 PMC6109056 30155510

